# The use of olanzapine as an antiemetic in palliative medicine: a systematic review of the literature

**DOI:** 10.1186/s12904-020-00559-4

**Published:** 2020-04-22

**Authors:** G. Saudemont, C. Prod’Homme, A. Da Silva, S. Villet, M. Reich, N. Penel, V. Gamblin

**Affiliations:** 1 Territorial Hub Pain Accompaniment Palliative care, Hospital center Antibes Juan-les-Pins, 107 avenue de Nice, F-06606 Antibes, France; 2 University Lille, CNRS, CHU Lille, Palliative care unit, F-59000 Lille, France; 3 ETHICS (Experiment, Transhumanism, Human Interactions, Care and Society) – EA 7446, Lille Catholic University, Lille, France; 4 Palliative care unit, Oscar Lambret center, 3 rue Frédéric Combemale, F-59020 Lille, France; 5 Direction of Research and Innovation, Oscar Lambret center, 3 rue Frédéric Combemale, F-59020 Lille, France; 6 Lille University Hospital and Medical School, F-59045 Lille, France

**Keywords:** Palliative medicine, Antiemetic treatment, Atypical antipsychotic, Olanzapine

## Abstract

**Background:**

Olanzapine is an atypical antipsychotic that has affinity for many central nervous system receptors. Its efficacy is supported by several studies in the prevention and treatment of chemotherapy-induced nausea and vomiting. No recommendations exist on the antiemetic use of olanzapine in the palliative care setting. The aim of this work is to complete the initial work of Fonte et al. published in 2015, to determine whether the literature supports the use of olanzapine as an antiemetic in palliative situations and, in practice, to propose a therapeutic schema adapted to the palliative setting.

**Methods:**

Systematic review of the literature according to the PRISMA criteria. We searched the PubMed, Cochrane, RefDoc, EMBase databases and the gray literature databases. The bibliographic search was conducted between November 2016 and August 2017.

**Results:**

Thirteen articles were included: 2 case studies, 3 case series, 3 retrospective studies, 2 prospective studies, 2 literature reviews. All studies concluded on the efficacy of olanzapine as an antiemetic in the palliative care setting. No serious adverse effects were reported. Based on the data from the literature review, we propose a therapeutic scheme adapted to the palliative care context.

**Conclusion:**

Action of olanzapine on many receptors and its tolerance profile make it an interesting antiemetic treatment in palliative medicine. But to date, studies are scarce and have a low statistical power. Further investigation is therefore needed to determine the benefit of this treatment in palliative care patients, compared to usual treatments.

## Background

Since the early 2000s, the antiemetic efficacy of olanzapine, a drug conventionally used as an antipsychotic, has been suggested [1]. Numerous studies and literature reviews have been conducted on its efficacy and safety profile for the prevention and treatment of chemotherapy-induced nausea and vomiting (CINV) [2–4].

In three recent systematic reviews the most frequently reported adverse event was drowsiness, which was mostly well tolerated by patients [5–7]. No severe side effects were described.

Some studies concluded on a greater efficacy of olanzapine as a crisis medication for CINV despite standard prophylaxis, over standard crisis medication (metoclopramide - Primpéran®).

The Multinational Association for Supportive Care in Cancer (MASCC) and the National Comprehensive Cancer Network (NCCN) recommend olanzapine for treating refractory CINV in addition to appropriate preventive treatment. NCCN also recommends its use in combination with a selective antagonist of the 5HT_3_ receptor and corticosteroids, or with corticosteroids alone, for the prevention of CINV [8, 9].

Our primary objective was to investigate whether these results could be generalized to the prevention and treatment of nausea and vomiting not induced by chemotherapy in a palliative care setting. We also aimed at supplementing and updating the non-systematic review published by Fonte et al. in 2015 [10], which focused mainly on the use of olanzapine in chemotherapy-induced nausea and vomiting.

First, we describe the characteristics of olanzapine, including its tolerance profile and receptor-binding ability, which is of interest in the palliative care setting. Secondly, the findings of a systematic literature review of olanzapine used in this indication are presented to provide an update on current knowledge. Finally, we propose a therapeutic schema adapted to the palliative setting.

### Olanzapine, an atypical antipsychotic

#### Overview on atypical antipsychotics

The efficacy of antipsychotics drugs stems from their action on the dopaminergic system, which plays a role in the regulation of emotional life, motivation control, modulation of perceptions and organization of adaptive behaviors. According to their side effects, antipsychotics can be classified as first-generation, which are frequently associated with extrapyramidal effects, and second-generation, which are atypical and better tolerated [11].

Atypical antipsychotics (AAPs) are those meeting the following criteria [12]:

- have low or no risk of triggering extrapyramidal effects at doses at which an antipsychotic effect is achieved,
- do not increase prolactin levels, or do it minimally,
- significantly reduce the positive and negative symptoms of schizophrenia, and
- have mood stabilizer properties.

They also have an atypical binding profile to brain receptors. Atypical agents have a greater in vitro affinity for serotoninergic 5HT_2_ and dopaminergic D_2_ receptors than first-generation antipsychotics [12]. The main representatives of second-generation antipsychotics are: olanzapine (Zyprexa®), clozapine (Leponex®), risperidone (Risperdal®), quetiapine (Xeroquel®), sertindole, ziprasidone (Zeldox®), aripiprazole (Abilify®), paliperidone (Xeplion®), lurasidone (Latuda®), asenapine (Sycrest®).

#### Olanzapine

### Pharmacology

- Pharmacodynamic properties

Olanzapine is a thienobenzodiazepine derivative and has a structure close to that of clozapine [11]. It binds to many types of dopaminergic (D_1_, D_2_, D_3_, D_4_, D_5_) and serotoninergic (5HT_2A / 2C_, 5HT_3_, 5HT_6_ and 5HT_7_) receptors, but its affinity for 5HT_2_ receptors - in particular 5HT_2A_ – is higher than that for dopaminergic receptors. Olanzapine is also an antagonist of the muscarinic M_1_, M_2_, M_3_, M_4_, M_5_ (contributing to reduce the risk of extrapyramidal effects), α1-adrenergic and histamine H1 receptors. Its affinity for α2-adrenergic, 5HT_1_, GABA (Gamma-AminoButyric Acid), β-adrenergic and benzodiazepine receptors is lower. Olanzapine also has a low antagonistic effect on N-Methyl-D Aspartate (NMDA) receptors [13, 14].

- Indications, dosages

Olanzapine is an antipsychotic agent, an antimanic and mood stabilizer indicated in the management of manic episodes, schizophrenia, and bipolar disorder [15]. It has also been indicated for delirium especially in palliative care [16, 17]. The usual dose ranges between 2.5 and 30 mg once daily, but some studies report the use of the maximum dose of 60 mg per day depending on the symptomatology, the treatment response and the tolerance [15, 18].

- Pharmacokinetic properties

The bioavailability of oral olanzapine is 80 to 90%, and the peak serum concentration is reached approximately 6 h after administration. After intramuscular administration, olanzapine is rapidly absorbed and the time to peak serum concentration is less than 45 min. While a few studies have focused on the intravenous (IV) and subcutaneous (SC) routes, no pharmacokinetic data have been reported [19, 20]. The plasma protein binding rate is about 90%. The elimination half-life of olanzapine is approximately 30 h, ranging from 20 to 70 h [13, 15]. Plasma equilibrium is reached within 5 to 7 days. Hepatic first-pass effect is important, with 40% of the administered dose metabolized before it enters the systemic circulation [13]. Olanzapine is mainly metabolized in the liver by conjugation and oxidation and the main metabolites, 10-N-glucuronide and 42-N-desmethyl olanzapine, have no known pharmacological activity. The main route of olanzapine elimination is the oxidative metabolism by CYP1A2, while CYP2D6 and CYP2D19 are minor pathways [12, 15]. The metabolites are then eliminated through urine (60%) and feces (30%) [13].

- The benefits of olanzapine as an antiemetic.

Antiemetic treatments act by blocking receptors that are specific to neurotransmitters involved in transmitting the emetic signal to the vomiting center. The main treatments can be classified according to the targeted receptors [21–23]:

- Prokinetics: these stimulate the motility of the upper digestive tract through several mechanisms of action:

o By activating 5HT4 serotonin receptors.

o By blocking 5HT3 serotonin receptors.

o By activating motilin receptors.

o By inhibiting the dopamine system.

- Dopamine antagonists: certain antipsychotic agents block dopamine D2 receptors located in the CTZ. All of these, except for haloperidol, have a broad spectrum of activity and also act on histamine, muscarinic, serotonin and/or alpha adrenergic receptors.

- Serotonin antagonists (5HT3): the 5HT3 receptors are located on the vagus nerve which sends signals to the vomiting center, on the enterochromaffin cells of the digestive tract, in the nucleus of the solitary tract and in the CTZ.

- Anticholinergic antihistamines: the first histamine receptor antagonists, known as piperazines, block H1 receptors in the vomiting center, in the vestibular nucleus and in the CTZ. Antimuscarinic activity also reduces mucous secretion.

- Anticholinergics: their ability to block muscarinic receptors relaxes the smooth muscles and reduces gastrointestinal secretion. They are particularly indicated in cases of malignant bowel obstruction.

- Neurokinin-1 receptor antagonists: their mechanism of action is based on their ability to inhibit the binding of substance P to the NK1 receptors in the digestive tract and in the vomiting center of the brain. The inhibitory action of olanzapine, in particular on the serotonin receptors 5HT_2_ and 5HT_3_ and the dopamine receptor D_2,_ explains its antiemetic activity.

The inhibitory action of olanzapine, in particular on the serotonin receptors 5HT_2_ and 5HT_3_ and the dopamine receptor D_2,_ explains its antiemetic activity. To a lesser extent, olanzapine is also a histamine and muscarinic receptor antagonist [13]. However, the mechanisms involved are not fully understood. The benefit of olanzapine, the only atypical antipsychotic to have antiemetic properties, is that it acts on a large number of receptors, while nausea and vomiting in a palliative situation are very often caused by multiple factors and may require the combination of several treatments, which increases the risk of drug interactions.

## Literature review

### Methods

We conducted this systematic review in accordance with the international PRISMA (Preferred Reporting Items for Systematic Reviews and Meta-Analysis) guidelines [24].

We aimed at identifying studies addressing these research questions:

1/ Does olanzapine have a role as an antiemetic in the palliative care setting?

2/ Is it possible to propose a therapeutic schema in the palliative care setting?

### Inclusion and exclusion criteria

We included articles meeting the following criteria: all study types, publication date not specified, written in English or French, studies carried out in adult patients in the palliative care setting and studies about the use of olanzapine as an antiemetic.

Studies on CINV, olanzapine used for other indications, including psychiatric and articles not available full length online were excluded from the review.

### Bibliographic search

We searched PubMed, Cochrane, RefDoc, ScienceDirect and EMBase databases and OpenGrey, Gray Literature Report and VigiPallia sites, which list the gray literature. We also consulted the site of the *French Society for Palliative Care (SFAP), national and international health agencies (HAS, WHO) and of the French National Agency for Medicines and Health Products Safety (ANSM).* The bibliographical references of the selected articles were examined to complete the search.

The following MeSH terms [25] were used in the search: *olanzapine, nausea, vomiting, antiemetic, palliative care, end of life, nausea, vomiting, emesis, antiemetic, palliative care, end of life*. The search algorithms for each database are presented in Supplementary Table 1.

The bibliographic search was conducted between November 2016 and August 2017.

### Selection of articles, collection and data analysis

We selected articles eligible for the review in three successive steps: review of the title, review of the abstract, and review of the full text. After reading and analyzing the selected articles, we extracted the following information: title, author(s), review, year of publication, country, methodology, study population; dosage used, main results.

## Results

1. Selection of articles

### Results of searches in databases

One researcher (G.S.) obtained 943 articles by querying the different databases and one additional article was included after reviewing the grey literature. No other reference was identified by examining selected articles.

### Article selection procedure

**Fig. 1:**
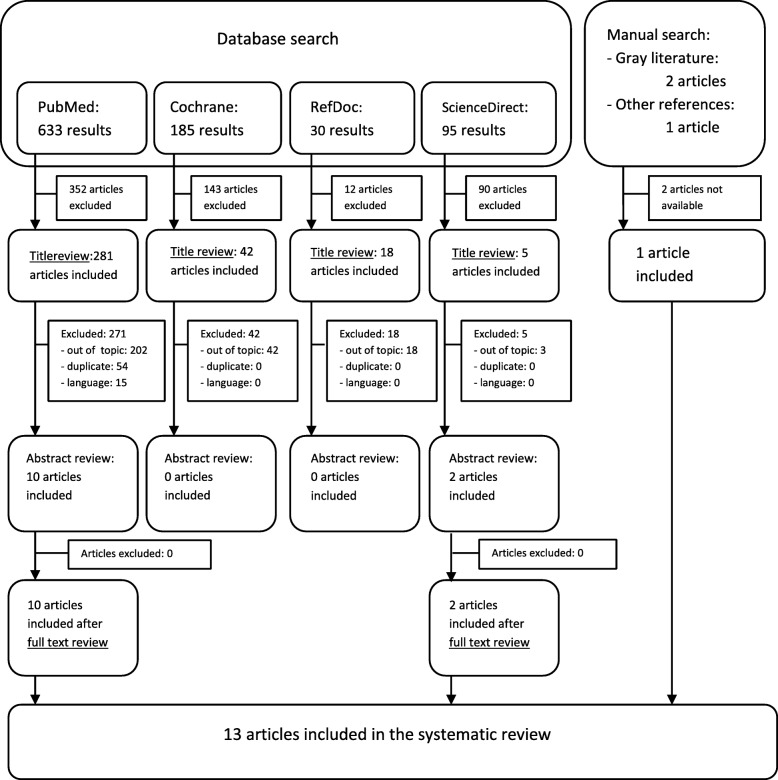
Flow diagram for the article selection procedure

Figure 1 presents the PRISMA flow chart with the selection procedure and the reasons for exclusion. We did not include EMBase search results because, out of the 123 research findings, only one involved the use of olanzapine as an antiemetic and the article was unavailable. In total, 13 articles were selected. The contents of the articles are summarised in Tables 1 and 2.

**Table 1:** Main results of the systematic literature review

Article	Type of study	Population	Results
Licup and Baumrucker, 2010 [26]	Literature review	Articles on the use of olanzapine as an antiemetic *N* = 4	2 articles on the use for chemotherapy-induced nausea and vomiting 2 articles on olanzapine in a palliative care setting Effective treatment No side effect reported
Fonte et al, 2015 [10]	Literature review	Article on the use of olanzapine as an antiemetic *N* = 22	15 articles on the use of olanzapine for the treatment of chemotherapy-induced nausea and vomiting 7 articles on olanzapine in a palliative care setting Effective treatment No significant side effects, except sleepiness reported in some studies
Felton et al, 2016 [27]	Literature review	Articles on the use of olanzapine in a palliative care population *N* = 12	2 articles on olanzapine for chemotherapy-induced nausea and vomiting 2 articles on olanzapine in a palliative care setting The other articles are on other indications Effective treatment No side effect reported
Passik et al, 2002 [28]	Prospective single centre study	Hospitalized patients with advanced cancer and refractory nausea and vomiting unrelated to radiotherapy or chemotherapy *N* = 15	In terms of nausea: dose-response efficacy (from 2.5 to 10 mg/day) In terms of quality of life: overall improvement only in patients treated at 5 mg/day No significant difference in side effects between patients without treatment and patients treated with different dosages
MacKintosch, 2016 [29]	Prospective single centre study	Hospitalized cancer patients with refractory nausea and vomiting *N* = 16	2 patients excluded due to treatment for less than 48 h Subjective evaluation: 13 patients (92%) reported improvement of symptoms 1 patient (7%) decided to stop treatment for excessive drowsiness
Kaneishi et al, 2012 [30]	Retrospective study	Patients hospitalized in the palliative care unit and treated with olanzapine for nausea and vomiting related to malignant bowel obstruction *N* = 20	Significant decrease in nausea score and frequency of vomiting after olanzapine treatment 18 patients (90%) reported a subjective improvement of nausea 2 patients (10%) reported excessive drowsiness 1 patient (5%) reported vertigo No decision to stop treatment despite symptoms
Atkinson, 2014 [31]	Retrospective study	Hospitalized cancer patients with refractory nausea and vomiting *N* = 4	Effective treatment Reduced use of rescue medication Reduced treatment cost No side effects reported
Kaneishi et al, 2016 [32]	Retrospective multicentre study	Patients with advanced cancer hospitalized in the palliative care unit and treated with olanzapine for nausea and vomiting *N* = 108	Doses ranged from 2.5 to 10 mg/day Average duration of treatment: 22 days (from 2 to 211 days) No efficacy or safety data reported.
Jackson and Tavernier, 2003 [33]	Case series	Hospitalized patients with cancer and neurological conditions and refractory nausea and vomiting *N* = 6	Effective treatment No side effects reported
Srivastava et al, 2003 [34]	Case series	Hospitalized cancer patients with refractory nausea and vomiting *N* = 2	Effective treatment No side effects reported
Atreya and Datta, 2016 [35]	Case series	Patients with advanced cancer hospitalized in palliative care unit with refractory nausea and vomiting *N* = 3	Effective treatment No side effects reported
Suzuki et al, 2014 [36]	Case study	1 patient with refractory nausea and vomiting after brain metastases from colorectal cancer	Effective treatment No side effects reported
Langley-Degroot et al, 2015 [37]	Case study	1 patient with refractory nausea and vomiting after dyskeratosis congenita	Effective treatment No side effects reported

**Table 2:** Summary of the dosages used and Previously Used

	Article	Dosage	Antiemetic treatments previously used
1	Licup and Baumrucker, 2010 [26]	- Seelines 4 and 10 - 2 other articles cited but no data	No data
2	Fonte et al, 2015 [10]	- See lines 4–6–7–9–10–12 - 1 other article cited but no data	No data
3	Felton et al, 2016 [27]	- See lines 4 and 6 - 2 other articles cited but no data	No data
4	Passik et al, 2002 [28]	3 dose groups: - 2.5 mg - 5 mg - 10 mg	No data
5	MacKintosch, 2016 [29]	5 mg/day	Haloperidol, promethazine, cyclizine, metoclopramide, ondansetron, levomepromazine, domperidone, prochlorperazine, quetiapine
6	Kaneishi et al, 2012 [30]	2.5 to 7.5 mg/day	No data
7	Atkinson, 2014 [31]	No data	No data
8	Kaneishi et al, 2016 [32]	2.5 mg to 10 mg/day	No data
9	Jackson and Tavernier, 2003 [33]	Stating dose2.5 mg/day Increment of 2.5 mg Maximum dose of 7.5 mg/day	Patient 1: haloperidol (effective but poor tolerance) Patient 2: promethazine, lorazepam Patient 3: No data Patient 4: dexamethasone, prochlorperazine, promethazine, haloperidol, lorazepam Patient 5: haloperidol, lorazepam Patient 6: No data
10	Srivastava et al, 2003 [34]	Stating dose 2.5 mg/day Increment of 5 mg/day Interdose of 5 mg possible	Patient 1: prochlorperazine, metoclopramide, dexamethasone, promethazine, doxepin, scopolamine, meclizine, haloperidol Patient 2: granisetron, lorazepam, metoclopramide, dexamethasone, haloperidol
11	Atreya and Datta, 2016 [35]	Stating dose 2.5 mg/day Increase up to 7.5 mg/day	Patient 1: dexamethasone, ondansetron, metoclopramide (stopped at introduction of olanzapine) Patient 2: metoclopramide, haloperidol Patient 3: dexamethasone, ondansetron, scopolamine butylbromide
12	Suzuki et al, 2014 [36]	Stating dose 1.25 mg/day	Metoclopramide, granisetron
13	Langley-Degroot et al, 2015 [37]	Starting dose 5 mg/day Increment of 10 mg/day	Initially metoclopramide, lorazepam, ondansetron, domperidone, cannabinoids

## Content of articles

## Discussion

### The place of Olanzapine as an antiemetic in palliative medicine

#### Efficacy

Each of the 13 articles included in this systematic review of the literature concluded that olanzapine is effective for the treatment of nausea and vomiting in patients with palliative disease, regardless of symptom aetiology. However, the differences in study methodologies and in the methods for evaluating symptoms prevent a rigorous comparison.

#### Applicability

In addition to its efficacy, several features make olanzapine an interesting treatment option in the palliative care setting.

- Pharmacokinetics

Olanzapine can be administered as a single, daily dose because of its long half-life that allows to cover a 24-hour period. This may facilitate patient compliance and reduce the risk of forgetfulness. In addition, it appears that, despite treatment for nausea and vomiting, rescue medications can be considered [6].

- Galenic forms

Olanzapine exists in several galenic forms, allowing flexibility in administration. The orodispersible tablet formulation is particularly suitable for patients experiencing nausea or vomiting, and represents a safer and more flexible option than haloperidol in the outpatient setting. In fact, oral forms of haloperidol [38] include tablets at doses of 1 or 5 mg, while the usual antiemetic dose starts at 0.5 mg. The oral solution is dosed at 0.1 mg per drop, but drop count is a source of error.

In the case of the treatment of symptomatic intestinal obstruction on non-resectable peritoneal carcinomatosis, scientific and medical societies recommend using haloperidol as first-line antiemetic treatment [39]. Oral intake in this context can be a source of discomfort for the patient, and the absorption of the drug is uncertain. In addition, in 2011, the National Agency for Drug Safety advised against the use of IV haloperidol due to cardiac risks [40, 41]. The Marketing Authorization (MA) for the injectable form of haloperidol relates exclusively to the intramuscular (IM) route, regardless of the indication.

Olanzapine exists as injectable form and its MA only applies to IM use. Studies on the IV or SC use for other indications, however, report a comparable efficacy and no significant side effects [19, 20].

In addition, pharmacological studies suggest a part of transmucosal absorption with orodispersible tablets, even if the proportion and its impact on the overall bioavailability are unknown. Olanzapine is detectable earlier in the plasma of patients treated with orodispersible tablets than of those treated with standard tablets. This could prove to be an asset in case of occlusive syndrome [42–44]. This hypothesis is supported by the results of the 2012 study by Kaneishi et al. [30], which involved patients with nausea associated with malignant bowel obstruction. One study also investigated a form of reconstituted olanzapine as suppositories [45], which would provide an additional usable route of administration.

- Tolerance profile

AAPs present a lower risk of adverse effects than first generation antipsychotics. Various explanations have been advanced, including the 5HT_2A_/D_2_ binding affinity ratio. The 5HT_2A_/D_2_ ratio is higher for AAPs than for first-generation antipsychotics [46], which explains the lower risk of extrapyramidal syndrome [47]. In addition, olanzapine is predominantly metabolised via cytochrome P1A2, and not via the other CYP450 isoenzymes [10], which also limits the risk of drug interaction. Molecules such as carbamazepine (Tegretol®) or fluvoxamine (Floxyfral®) may, however, interact with CYP1A2. Similarly, enzyme inducer tobacco, can also modify the metabolism of olanzapine by interacting with cytochromes [11, 47, 48].

Finally, the antiemetic dose used, generally ranging from 2.5 to 10 mg/day, is lower than that used in psychiatry, which also explains the lower risk of adverse effects [10].

#### Adverse effects/side effects

The treatment of CINV involves low doses of olanzapine over shorter periods of time, leading to few side effects. Most commonly reported event is drowsiness [29, 30]. However, this symptom remains difficult to assess in the palliative care context, where the causes can be multiple, mostly iatrogenic and disease progression. No other side effects of olanzapine have been reported in the studies included in this review of the literature.

In the psychiatric setting, olanzapine is used at higher doses and over longer periods of time. The extrapyramidal syndrome, consisting of acute dystonia, akathisia, parkinsonism and tardive dyskinesia [49], can occur at doses over 20 mg/day. However, the risk of developing a Parkinsonian syndrome or akathisia is, respectively, three times and twice lower than that with haloperidol at usual doses [47]. Long-term use of olanzapine can lead to metabolic side effects such as increased appetite and weight gain, which may be beneficial in the palliative care setting [50]. Lipid and glycaemic imbalances can also be observed during long-term treatment, which is why olanzapine is contraindicated in cases of diabetes in some countries [51]. There is a risk of QT prolongation or of cardiac rhythm disturbances, but these effects are rare [17, 52].

There is also a risk of decreasing the epileptogenic threshold and the neuroleptic malignant syndrome, but these are less common than with first-generation antipsychotics [10, 47].

Other side effects have been reported in this setting, of varying frequency and intensity, such as headache, drowsiness, restlessness, insomnia, dry mouth, constipation, orthostatic hypotension [47, 52]. Finally, an asymptomatic elevation of transaminases (up to 3 times the normal level) has been reported in 2% of patients [47, 52].

Contraindications to olanzapine are hypersensitivity to the active substance or to any of the excipients, and patients at risk of acute angle-closure glaucoma (AACG) [17]. In a statement of March 9, 2004, the ANSM (French National Agency for Medicines) also recommended the utmost caution and advised against the use of olanzapine in elderly patients with dementia due to a threefold higher incidence of stroke [53].

### Bias of the study

#### Intrinsic bias

The scarcity of published articles on the use of anti-emetic olanzapine in the palliative care setting is an obvious bias of this systematic review of the literature. In addition, the disparities in the methodology of the articles and the heterogeneity of outcomes used do not allow a rigorous analysis. Publication bias must also be considered, which may distort the effects of olanzapine. It is in order to limit this risk of bias that the gray literature has also been considered [54].

### Bias of the analysed studies

Most of the articles included in this review have low statistical power. Case studies and series include, by definition, low numbers of patients and present a selection bias. In the studies included in this review, symptoms were not objectively and reproducibly evaluated, with investigators measuring treatment efficacy based on patient-reported relief and clinical examination.

Retrospective studies also have a selection bias by definition. Two of the included studies concerned a small number of patients and, the third, a 2016 study by Kaneishi et al [32], included a larger number of patients but did not mention the efficacy or tolerance of the treatment.

The evaluation of treatment efficacy among studies is not standardised. In the 2012 study by Kaneishi et al [30], the outcome was the degree of severity of symptoms on a scale, while in Atkinson’s 2014 study [31], the evaluation of symptom improvement was subjective and the secondary outcomes were the use of treatment in situations of acute crisis and the daily cost of treatment.

The two prospective studies included in this review included few patients and neither were randomised nor did they include a control group. The evaluation of treatment efficacy in MacKintosch et al’s study was done using subjective criteria [29]. The study by Passik et al [28] provided a more complete assessment of symptoms with subjective assessment and objective evaluation using quality of life scales. The three reviews of the literature included are not systematic and their methodology was non-reproducible. Finally, only one study included an assessment of the quality of life of patients [28].

### In practice

Based on the study of pharmacology and the review of recent literature, despite a very low level of evidence, we suggest some approaches to use olanzapine in a palliative care setting for the treatment of nausea and vomiting refractory to other antiemetics [55] according to the scheme proposed below. This recommended treatment scheme is empirical and not based on prospective clinical trials.

Indications:

-the treatment of nausea and/or vomiting refractory to two previous lines of antiemetics, whatever the aetiology.

-in patients with the ability to take oral therapy.

Associated treatments:

Olanzapine may be combined with non neuroleptic antiemetic treatments.

For patients already treated with neuroleptics for nausea/vomiting, we recommend replacing this treatment with olanzapine, and not combining the two, to avoid side effects.

Contraindications:

Contraindications include those reported by VIDAL, ie hypersensitivity to the active substance or patients at risk of AACG (acute angle-closure glaucoma). In patients with a high risk of stroke or seizure, the risk/benefit ratio should be carefully evaluated.

Starting dose and galenic formulation:

We suggest a starting dose of 5 mg per day, preferably in the evening because of the risk of drowsiness, or at 2.5 mg per day in frail elderly patients.

Interdoses of 2.5 mg are possible once a day.

Dose adaptation:

In case of insufficient efficacy and good treatment tolerance, we propose to increase the dose in increments of 2.5 mg up to 10 mg per day.

Surveillance, side effects:

The surveillance will be clinical with an evaluation of the induced drowsiness, search for vertigo, particularly in patients who are still able to stand, and monitoring of neurological examination to detect an extrapyramidal syndrome.

## Conclusion

Olanzapine is an atypical antipsychotic that has antiemetic activity due to its affinity for many central nervous system receptors, particularly to the chemoreceptor trigger zone and to the vomiting center. Its antiemetic use in the prevention and treatment of CINV is recommended as second or even first-line treatment in the oncology setting by MASCC and NCCN.

The published studies on the use of olanzapine as antiemetic treatment in the palliative setting reveal an efficiency and a good tolerance of this treatment, which is also available in several galenic forms adapted to several modes of administration, in particular the orodispersible form. We propose the use of olanzapine in the palliative setting in the event of nausea or vomiting refractory to the usual antiemetic treatments at a dose of 5 mg per day with possible 2.5 mg interdoses.

Contraindications to its use are hypersensitivity to the active substance and the risk of AACG, and patients at high risk of stroke or seizure.

Two recent studies published in 2019, larger (*n* = 30 and *n* = 40) than those listed in our review of the literature, confirmed the safety and efficacy of olanzapine as an antiemetic for patients with late-stage cancer not receiving chemotherapy or radiotherapy [56, 57]. However, only Navari’s research was a randomized, double-blind, placebo-controlled study. To date, studies are scarce and have a low statistical power and therefore more prospective randomized controlled trials are needed to determine the benefit of this treatment in palliative care patients, compared to placebo or usual treatments as prokinetics, 5-HT3 antagonists or NK1 antagonists. Due to these patients’ frailty, it would also be wise for these studies to use the lowest possible starting dose (2.5–5 mg per day). The quality of life of patients should also be considered to evaluate and recommend their use as first-line treatment in a palliative care setting.

## Supplementary information

**Additional file 1: Supplementary Table 1.** Search algorythms used for each database.

## Data Availability

The datasets used and/or analysed during the current study are available from the corresponding author on reasonable request.

## Abbreviations

CINV: Chemotherapy-induced nausea and vomiting
MASCC: Multinational association for supportive care in cancer
NCCN: National comprehensive cancer network
AAPs: Atypical antipsychotics
GABA: Gamma-amino butyric acid
NMDA: N-methyl-D aspartate
IV: Intravenous
SC: Subcutaneous
PRISMA: Preferred reporting items for systematic reviews and meta-analysis
MA: Marketing authorization
IM: Intramuscular
AACG: Acute angle-closure glaucoma
ANSM: French national agency for medicines
CNIL: Commission nationale informatique et liberté
