# Supplementary material for: The use of olanzapine as an antiemetic in palliative medicine: a systematic review of the literature
Source: BMC Palliat Care. 2020 Apr 22;19:56. doi: 10.1186/s12904-020-00559-4 (PMC7178955; doi:10.1186/s12904-020-00559-4)
Supplement: Supplementary file 1 — Additional file 1: Supplementary Table 1. Search algorythms used for each database. [file 12904_2020_559_MOESM1_ESM.docx]

**Supplementary Table 1.** Search algorythms used for each database.

| **Database** | **Search algorythm** |
| --- | --- |
| **Pubmed** | Olanzapine AND palliative AND (nausea OR vomiting OR emesis OR antiemetic)  Olanzapine AND “end of life” AND (nausea OR vomiting OR emesis OR antiemetic)  Olanzapine AND palliative  Olanzapine AND “end of life”  Olanzapine AND (nausea OR vomiting OR emesis OR antiemetic) |
| **Cochrane** | Olanzapine AND palliative  Olanzapine AND “end of life”  Olanzapine AND (nausea OR vomiting OR emesis OR antiemetic) |
| **Refdoc** | Olanzapine AND palliative  Olanzapine AND “end of life”  Olanzapine AND (nausea OR vomiting OR emesis OR antiemetic) |
| **Science Direct** | Olanzapine AND palliative AND (nausea OR vomiting OR emesis OR antiemetic)  Olanzapine AND “soins palliatifs”  Olanzapine AND (nausée OR vomissement OR antiémétique) |
| **EMBase** | +Olanzapine [nausée] [vomissement] [antiémétique] [palliatif] |
